# Cerebral Desaturation Events During Shoulder Arthroscopy in the Beach Chair Position

**DOI:** 10.5435/JAAOSGlobal-D-19-00007

**Published:** 2019-08-02

**Authors:** Dane H. Salazar, William J. Davis, Nezih Ziroğlu, Nickolas G. Garbis

**Affiliations:** From the Department of Orthopaedic Surgery and Rehabilitation (Dr. Salazar, Davis, and Garbis), Loyola University Health System, Maywood, IL; and Orthopaedics and Traumatology Clinic (Dr. Ziroğlu), Bakirkoy Education and Research Hospital.

## Abstract

The beach chair position (BCP) is commonly used position in upper extremity surgery. Although there are many advantages to surgery in this position, there are also potential drawbacks and described complications including devastating neurologic outcomes. The etiology of these complications is postulated to be due to the gravitational effects of the seated position leading to cerebral hypoperfusion. We review the current literature on intraoperative cerebral monitoring and neurocognitive complications with shoulder surgery performed in the BCP. A previous systematic review estimated the incidence of neurocognitive complications after surgery in the BCP to be 0.004%. However, the true incidence is unknown and is likely much more common. Reports of neurologic complications have revealed a need for heightened vigilance, alternative anesthesia techniques, and improved monitoring. Methods for monitoring have included near-infrared spectroscopy, a measurement of cerebral oximetry shown to reliably detect cerebral hypoperfusion. In this literature review, we sought to update the incidence of intraoperative cerebral desaturation events (CDEs) to investigate the relationship of CDEs to neurocognitive complications and to review recent reported cases of neurocognitive complications. Existing literature suggest that accurate intraoperative monitoring of cerebral perfusion may improve patient safety.

The beach chair position (BCP) is commonly used position in upper extremity surgery. Compared with the lateral decubitus position, the BCP provides anatomic positioning of the shoulder, reduces risk of brachial plexus injury, and improves airway access.^1^ Although there are many advantages to surgery in this position, there are also potential drawbacks and described complications with semiupright positioning. Devastating neurologic outcomes have been reported, including stroke, brain death, vision loss, and death.^1,2^ The etiology of these complications is speculative but is postulated to be due at least in part to a hydrostatic gradient between the heart and the brain created by the gravitational effects of the seated position leading to cerebral hypoperfusion. This hydrostatic gradient can be substantially affected by the practice of hypotensive anesthesia, which has been used to reduce intraoperative bleeding.^1,2^ Diminished bleeding aides with improved intraoperative visibility, especially with arthroscopic surgery.^2^ However, intentional hypotensive anesthesia can lead to cerebral hypoperfusion and thus decrease the safety of surgery in the BCP.^2^ It is theorized that sustained hypoperfusion may cause ischemia, which rarely has lead to devastating permanent neurocognitive complications in previously healthy patients.^1–3^ We review the current literature on intraoperative cerebral monitoring and neurocognitive complications with shoulder surgery performed in the BCP.

## Proposed Pathoanatomy

Semiseated and sitting patient positioning during surgical procedures has been associated with cerebral hypoperfusion.^4^ Changes in cerebral perfusion pressure occur secondary to hemodynamic fluctuations as the patient is transitioned from supine to upright.^5^ In normal physiology, baroreceptors increase firing of the sympathetic nervous system and decrease firing of the parasympathetic nervous system to maintain blood pressure when a person moves from supine to upright.^6^ In anesthetized patients, the autonomic nervous system response is dampened by the vasodilating effects of intravenous and inhaled anesthetics.^4^ When a patient is maneuvered from supine to sitting, decreases in cerebral perfusion pressures have been detected and can lead to intraoperative cerebral desaturation events (CDEs) and potentially cerebral ischemia.^2,7^ The overestimation of blood pressure at the level of the brain is caused by “the waterfall” effect.^8^ The seated position creates a hydrostatic column of blood from the heart to the brain. As blood flows vertically, there is a reduction in arterial pressure directly related to the weight of the fluid column. This physiologic phenomenon explains the potential danger of intentional hypotensive anesthesia in the sitting position, as noninvasive blood pressure monitoring at the brachial artery will overestimate the blood pressure at the level of the brain.^2,4,8^ For this reason, there has been an investigation into more accurate ways of monitoring intraoperative blood pressure and brain perfusion for surgeries performed in the seated position. The use of cerebral oximetry using near-infrared spectroscopy (NIRS) to monitor the adequacy of cerebral perfusion and to guide intraoperative interventions in shoulder surgery has become more common over the last decade.^1,3,4,9,10,11,12,13,14,15,16,17,18,19,20,21,22,23,24,25,26,27,28,29,30,31,32^

## Neurocognitive Complications

Although seemingly rare, several case reports and small case series have been published on catastrophic neurocognitive complications in previously healthy individuals undergoing shoulder surgery in the BCP.^16,20,33,34,35,36^ A previous systematic review estimated the incidence of neurocognitive complications after surgery in the BCP to be 0.004%.^1^ However, the true incidence is unknown and is likely much more common, given the fact that once a case report of a complication is published in the literature, future case reports are unlikely to be submitted/published unless it adds something new or substantial to the topic. Pohl and Cullen^35^ reported four previously healthy middle-aged patients at extremely low risk of cerebrovascular events who sustained major brain injury during shoulder surgery in the upright position. Bhatti and Enneking^3^ described a case of acute postoperative vision loss and ophthalmoplegia attributed to intraoperative hypotension.^33^ Garnaud et al^16^ reported a case of cranial nerve VII and XII palsy manifested by a Horner sign, paretic dysarthria, and a swallowing disorder that presented on recovery from anesthesia. All symptoms were ipsilateral to the surgical shoulder. Kocaoglu et al^20^ reported a 52-year-old woman who underwent an arthroscopic rotator cuff repair and developed visual symptoms that lasted 48 hours. The authors noted that intraoperatively, the mean arterial pressure decreased below 65 mmHg for 5 minutes. This case served as an impetus for the authors to use NIRS monitoring during future surgeries in the BCP. Murphy et al^4^ briefly described a patient who experienced postoperative transient delirium after a thirty-four-minute intraoperative CDE during arthroscopic shoulder surgery. Friedman et al attempted to quantify the prevalence of intraoperative cerebrovascular events during shoulder surgery in the BCP. They surveyed 287 members of the American Shoulder and Elbow Surgeons. They had a 32% response rate and found that most of these surgeons averaged in excess of 300 shoulder cases annually. Most of these cases were arthroscopic, and the patient position is primarily beach chair. The total number of beach chair position surgeries was estimated between 173,370 and 209,628, and lateral decubitus position surgeries were estimated between 64,597 and 100,855.^37^ The overall rate of intraoperative cerebrovascular event was 0.00291% (8/274,225). All cerebrovascular events were associated with surgeries in the BCP. The rate in the BCP ranged from 0.00382% (8/209,628) to 0.00461% (8/173,370).^37^ Although uncommon, perioperative cerebral ischemic accidents are potentially devastating for patients, their families, and the healthcare professionals involved. These events have tremendous economic, social, professional, and medicolegal implications, with perioperative stroke being particularly morbid. Perioperative stroke has a mortality rate of 60% versus 15% to 46% for stroke in general.^38,39^

## Intraoperative Cerebral Perfusion Monitoring

Methods for monitoring have included electroencephalography, invasive blood pressure monitoring at brain level, and cerebral oximetry using NIRS. NIRS has become exceptionally popular and has been used extensively because of noninvasive nature, relatively low cost, ease of use, and ease of access. NIRS is a measurement of cerebral oximetry that has been shown to strongly correlate with middle cerebral artery flow velocity and to reliably detect cerebral hypoperfusion.^9^ NIRS is noninvasive and readily available at most hospitals, as this technology is often used intraoperatively for procedures with a high risk of neurologic complications including cardiac, intra-abdominal, neurologic, and vascular surgeries.^3,40^ Twenty recent publications have reported on intraoperative perfusion monitoring of patients undergoing shoulder arthroscopy in the BCP. The incidence of CDEs among these articles is presented in Table 1. The minimum incidence of intraoperative CDEs was 0% in a cohort of 50 patients,^30^ and the maximum incidence was 57% in a cohort of 98 patients.^24^ Although there was wide variability in CDE incidence, the studies were consistent with respect to their definition of a CDE. Most articles used a decrease in rSO_2_ of 20% or more from baseline and/or an absolute rSO_2_ value less than or equal to 55% to define a CDE. However, there was variability in determination of baseline values. Some baselines were recorded before oxygenation, some after noninvasive oxygen supplementation, and others after intubation. Only one of the 21 studies reported a clinically significant neurocognitive event; Murphy et al^26^ reported a patient experiencing transient delirium after a 34-minute intraoperative CDE. This neurocognitive deficit lends to the benefit of having intraoperative cerebral oxygenation monitoring, as NIRs provide a method of detecting desaturation that would not otherwise be detected with standard monitoring.^17^ However, when the incidence of CDEs compared with the incidence of neurocognitive deficits is assessed with the cost and availability of this advanced monitoring, the clinical utility of NIRS may be limited. Future studies with standardized use of NIRS and standardized monitoring of neurocognitive outcome are needed to further assess clinical utility.

**Table 1:**
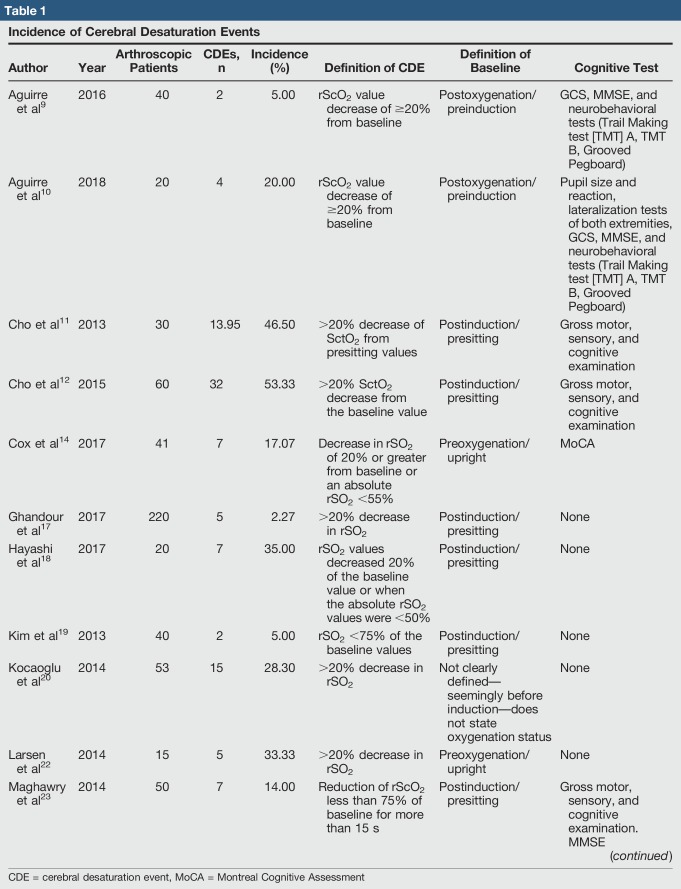

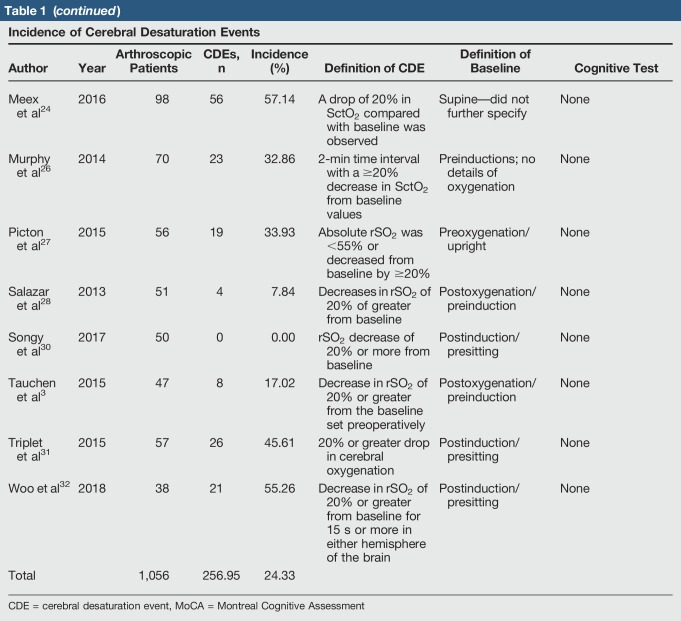
Incidence of Cerebral Desaturation Events

## Correlation Between Intraoperative Cerebral Perfusion Deficits and Postoperative Neurocognitive Deficits

Multiple previous investigations have attempted to establish a correlation between intraoperative hypoperfusion and postoperative neurocognitive deficits. In a previous study of 50 consecutive patients undergoing shoulder arthroscopy in the BCP, regional cerebral tissue oxygen saturation (rSO2) was monitored intraoperatively using NIRS. The Repeatable Battery for the Assessment of Neuropsychological Status was administered to each patient pre- and postoperatively, as its sensitivity allows detection of mild cognitive impairment and it is validated to assess postsurgical cognitive changes. The incidence of intraoperative CDEs was 18% (9/50). They found no statistical significance in pre- versus postoperative Repeatable Battery for the Assessment of Neuropsychological Status either in composite scores or any of the subindices in either group.^29^ Cox et al used NIRS to monitor cerebral oxygenation saturation in 41 consecutive patients undergoing arthroscopic shoulder surgery in the BCP. Patients were randomized into two groups, and anesthetists aware of or blinded to NIRS data. The Montreal Cognitive Assessment, which has been shown to detect mild cognitive deficits, was used to assess cognitive function preoperatively, immediately postoperatively, and at 2 and 6 weeks postoperatively. Overall, 7 patients (17.5%) experienced a CDE, 5 (25%) in the aware group, and 2 (10%) in the blinded group. There was no significant difference in Montreal Cognitive Assessment scores between the aware and blinded groups preoperatively (27.9.1 versus 28.2; *P* = 0.436), immediately postoperatively (26.1 versus 26.2; *P* = 0.778), 2 weeks postoperatively (28.0 versus 28.1; *P* = 0.737), or 6 weeks postoperatively (28.5 versus 28.4; *P* = 0.779) (Cox 29396097).^14^ Laflam and colleagues performed a comparative investigation of 109 patients undergoing shoulder surgery in the BCP and 109 patients in the lateral decubitus position using regional cerebral oxygen saturation (rScO_2_) monitored with NIRS. Psychometric testing, with the National Institutes of Health Stroke Scale, Rey Auditory Verbal Learning Test, Controlled Oral Word Association Test, Symbol Digits Modalities Test, Trail Making B, and Grooved PegBoard Test, was performed before surgery and then 7 to 10 days and 4 to 6 weeks after surgery. A composite cognitive outcome was determined as the Z-score. Serum biomarkers that are associated with brain injury—S100β, neuron-specific enolase, and glial fibrillary acidic protein—were measured at baseline, after surgery, and on postoperative day 1. After adjusting for baseline composite cognitive outcome, there was no difference in Z-score 7 to 10 days (*P* = 0.530) or 4 to 6 weeks (*P* = 0.202) after surgery between the BCP and the LDP groups. There was no difference in serum biomarker levels between the two position groups.^21^

Aguirre et al^10^ performed a study to assess the effect of general anesthesia and controlled hypotension on cerebral saturation (rScO_2_), cerebral blood flow, and neurobehavioral outcomes in 40 patients undergoing shoulder surgery in BCP. They collected neurologic and neurobehavioral tests including the Trail Making Tests A and B and the Grooved Pegboard test. rScO_2_ was monitored using NIRS, and cerebral blood flow was monitored using Doppler of the middle cerebral artery. The authors found that the incidence of CDEs was 25%. There were no neurologic deficits, but patients with CDEs performed worse on the Trail Making Test B and the Grooved Pegboard test 24 hours after surgery compared with patients without CDEs (*P* = 0.001).^10^

Our previous review highlighted the abundance of literature reporting previously healthy patients who develop neurologic complications after arthroscopic shoulder surgery in the BCP.^1^ The BCP has been implicated as a source of cerebral hypoperfusion and subsequent cerebral ischemia. The exact etiology of central nervous system injuries is incompletely understood and is thought to be multifactorial. However, hypoperfusion is thought to be the determining factor of poor neurologic outcome.^1,3^ Reports of neurologic complications have revealed a need for heightened vigilance, alternative anesthesia techniques, and improved monitoring.^1^ In this literature review, we sought to update the incidence of intraoperative CDEs, to investigate the relationship of CDEs to neurocognitive complications, and to review recent reported cases of neurocognitive complications, all in patients who have undergone arthroscopic shoulder surgery in the BCP.

## Summary

Neurocognitive complications after shoulder arthroscopy in the BCP are exceedingly rare but potentially catastrophic events that may affect patients without preexisting cerebrovascular risk factors. A previous systematic review of 24,701 cases reported the overall incidence of neurologic deficits after arthroscopy in the upright position to be 0.004%.^1^ The severity, frequency, and duration of hypoperfusion that cause cerebral ischemia and subsequent neurocognitive deficits have yet to be defined in arthroscopic shoulder surgery. Multiple previous reports have failed to establish a correlation between intraoperative CDEs and postoperative neurocognitive deficits. Large prospective clinical studies and further preclinical research are still needed to understand the clinically significant thresholds of magnitude, duration, and frequency of intraoperative CDEs to clearly establish a relationship with postoperative neurocognitive complications. Such large studies are also needed to further illuminate modifiable patient risk factors and to establish a system of sensitive, safe, and costeffective cerebral perfusion monitoring. Existing literature suggests that accurate intraoperative monitoring of cerebral perfusion may improve patient safety.
